# Coupling of coastal activity with tidal cycles is stronger in tool-using capuchins (*Cebus capucinus imitator*)

**DOI:** 10.1098/rsos.230355

**Published:** 2023-09-20

**Authors:** Zoë Goldsborough, Margaret C. Crofoot, Shauhin E. Alavi, Evelyn Del Rosario-Vargas, Sylvia F. Garza, Kate Tiedeman, Brendan J. Barrett

**Affiliations:** ^1^ Department for the Ecology of Animal Societies, Max Planck Institute of Animal Behavior, Konstanz, Germany; ^2^ Department of Biology, University of Konstanz, Konstanz, Germany; ^3^ Center for the Advanced Study of Collective Behavior, University of Konstanz, Konstanz, Germany; ^4^ Smithsonian Tropical Research Institute, Ancon, Panama

**Keywords:** tool use, tidal cycles, foraging, activity patterns, islands

## Abstract

Terrestrial mammals exploiting coastal resources must cope with the challenge that resource availability and accessibility fluctuate with tidal cycles. Tool use can improve foraging efficiency and provide access to structurally protected resources that are otherwise unavailable (e.g. molluscs and fruits). To understand how variable accessibility of valuable resources shapes behavioural patterns, and whether tool use aids in the efficient exploitation of intertidal resources, we compared the relationship between tidal cycles and activity patterns of tool-using versus non-tool-using groups of white-faced capuchin monkeys on Jicarón Island in Coiba National Park, Panama. Although tool use on Jicarón is localized to a small stretch of coast (approx. 1 km), all coastal groups forage on intertidal resources. Using more than 5 years of camera trap data at varying distances from the coast, we found that capuchins on Jicarón showed increased coastal activity during specific parts of the tidal cycle, and that this relationship differed between tool-using and non-tool-using groups, as well as between seasons. Activity patterns of tool-using capuchins were more strongly and consistently tied to tidal cycles compared with non-tool-users, indicating that tool use might allow for more efficient exploitation of tidal resources. Our findings highlight the potential of tool use to aid niche expansion.

## 1. Introduction

Animals that are dietary generalists are buffered against the negative consequences of environmental change and fluctuating resource availability [1]. Dietary generalists have been shown to be more innovative and better at spatial reasoning than specialist congeners, helping them to access patchy resources [2,3], and have also fared better than specialists in the face of anthropogenic change [4]. Maritime mammals (*sensu* [5]) are one example of dietary generalists: these are populations of mammalian foragers who exploit resources in the intertidal zones, despite intertidal areas being an unlikely selective pressure in their evolutionary past, and who typically also forage in other habitats.

While all animals must deal with spatio-temporal variability in choosing when and where to forage, terrestrial foragers exploiting intertidal resources face additional challenges. Tidal cycles are asynchronously cyclical: tidal peaks occur predictably, but the timing of low and high tides shifts daily and the magnitude of tidal change is affected by a myriad of interacting processes. Resource accessibility also varies depending on season and weather. Different prey species may show intra-annual variability in abundance due to migration or breeding patterns [6,7]. Prey accessibility can be limited by sunlight and temperature, which can heat substrates to greater than 45°C and impact tidal pool salinity causing prey to conceal themselves or move towards the ocean [8–10]. Further, the intertidal zone in tropical regions is often exposed and hot, which potentially puts individuals with reduced adaptation to cope with hot substrates at risk of thermal stress when traversing the intertidal zone. Although a taxonomically diverse set of mammalian taxa are known to exploit intertidal resources (e.g. primates [11,12], carnivores [13–15] and rodents [16]), it is unclear *how* this variable accessibility of valuable resources affects the daily activity patterns of these maritime mammals.

### 1.1. Tool use improves efficiency of intertidal foraging

The importance of intertidal resources and the extent to which maritime mammals alter their activity patterns to tidal cycles may depend on their ability to exploit the resources in these dynamic habitats. Intertidal resources, such as marine invertebrates and fruits with ocean dispersed seeds, are physically protected and therefore require time-consuming processing (e.g. bivalves, crabs). One way in which maritime mammals could exploit intertidal resources more efficiently and access resources that are otherwise inaccessible to them is by using tools. Tool use facilitates more efficient resource consumption through faster processing, and provides access to structurally protected foods [17], and as such allows animals to expand their effective environment. Rocky intertidal areas exposed by low tides provide a wealth of anvil and hammerstone material, in the form of bedrock, driftwood and smooth stones.

A behaviour is considered tool use when an animal manipulates an object that is not part of their own body in order to reach a useful outcome [18,19]. Examples of animal tool use in foraging contexts are widespread, ranging from chimpanzees (*Pan troglodytes*) using sticks for termite fishing [20] and robust capuchins (*Sapajus* spp*.*) using stone tools to crack nuts [21,22], to dolphins (*Tursiops* sp*.*) protecting their rostra with sponges while foraging on the seafloor [23] and New-Caledonian crows (*Corvus moneduloides*) crafting and using hooked as well as unhooked twig tools to forage on embedded insects [24].

### 1.2. Maritime mammals face challenges in timing foraging with tidal cycles

How intertidal foraging impacts a maritime mammal's behaviour depends on the importance of these resources in their diets. Carlton & Hodder [5] distinguish between opportunistic and obligate foraging in the intertidal zone. Opportunistic omnivores can exploit the intertidal zone whenever it is advantageous to them. By contrast, obligate reliance on intertidal resources can be a result of seasonal or systematic impoverishment of terrestrial resources which drives maritime mammals to intertidal resources as an essential addition to their diet [5], making intertidal resources a type of fallback food [25,26]. Opportunistic exploitation of the intertidal zone has different effects on maritime mammals' activity patterns. An example of opportunistic maritime mammals are Japanese macaques (*Macaca fuscata*), who feed on seafood and only show intertidal foraging linked to tidal cycles in months when terrestrial resources are scarce [27]. By contrast, black legged kittiwakes (*Rissa tridactyla*)—who are more obligate maritime foragers as they systematically rely on intertidal resources—choose to forage in the intertidal zone and leave their chicks unattended when low tide overlaps with their nesting period [28]. Furthermore, island-living arctic foxes (*Alopex lagopus*), whose diet is dominated by fish caught in tidal pools, shape their daily activity pattern around the tides: they forage at low tide and sleep at high tide [29]. Animals that exploit resources in the intertidal zone may thus benefit from adjusting their daily activity to the tidal cycles.

### 1.3. The role of tool use in intertidal exploitation

Tool use might aid intertidal exploitation, as many intertidal resources are structurally protected (e.g. snails, bivalves, coconuts). An example of animals using tools to forage in intertidal zones with greater efficiency are bearded capuchins (*Sapajus apella* and *Sapajus libidinosus*) in mangroves. These capuchins exploit intertidal resources, including crabs and snails, with and without tools [30]. Notably, while crabs can be eaten without using tools, it appears that snails can only be consumed by tool-using capuchins. Island-living Burmese long-tailed macaques (*Macaca fascicularis aurea*) exploit both terrestrial resources (palm nuts) and tidal resources exposed at low tide, such as oysters, by using stone tools [11]. Intertidal resources may be of differing importance to tool-using and non-tool-using maritime mammals, as tool use can provide access to novel foraging niches [31] or otherwise inaccessible items [32]. Thus, tool-using maritime mammals may access more marine prey items than non-tool-using mammals. Alternatively, tool-users might have *less* need for intertidal resources than non-tool-users as they can access more nutrient-rich foods inland as well. In this case, non-tool-using maritime mammals might exploit intertidal resources to a greater extent as they have fewer terrestrial resource options.

### 1.4. Coiban capuchins are tool-using and non-tool-using maritime mammals

White-faced capuchins in Coiba National Park, Panama (hereafter Coiban capuchins), have been reported to habitually conduct first-order (e.g. pounding of coconuts on anvils [33]) and second-order tool use (e.g. hammerstone and anvil stone tool use [34,35]). This habitual tool use behaviour provides the opportunity to explore: (i) if capuchins adjust their patterns of activity to coincide with the tides and (ii) whether this relationship differs between tool-using and non-tool-using capuchins. White-faced capuchins are exploratory dietary generalists who rely heavily on extractive foraging to access structurally protected resources in varied neotropical forests [36–38]. Many of these extractive foraging behaviours are socially learned [39–41]. Despite decades of studies across multiple field sites [42,43], habitual stone tool use by white-faced capuchins has only been documented in at least one group of capuchins on the island of Coiba [35] and one group on the island of Jicarón [34] in Coiba National Park, located approximately 30 km off the Pacific coast of Panama. Recently, stone tool use was also described in an urban population of *Cebus albifrons* in Ecuador [44]. Coiban capuchins use hammerstone and anvil tool use to access a variety of food items, including sea almonds (*Terminalia catappa*), coconuts (*Cocos nucifera*), Halloween crabs (*Gecarcinus quadratus*), palm fruits (*Bactris major* and *Astrocaryum* spp*.*), hermit crabs (*Coenobita compressus*), nerite snails (*Nerita* sp*.*) and other freshwater molluscs. Coiban capuchins differ from well-studied mainland populations in showing increased terrestrial activity, likely due to the lack of mammalian predators on the island [45] and live at high population densities [34,46]. Additionally, both Coiba and Jicarón have lower plant richness than similar mainland ecosystems [47,48]. On Jicarón, this tool use tradition is highly localized along the coast and mostly limited to one social group [34], despite similar ecological circumstances and no physical barriers between the tool-using capuchin group and other groups of non-tool-using capuchins on the island. Further, both tool-using and non-tool-using capuchins on Jicarón are maritime mammals—that forage on (structurally protected) marine prey items along the coast and in the intertidal zone, such as crabs and snails [34] (electronic supplementary material, video S1). Coiba National Park experiences mixed tides; two low tides of different heights occur every approximately 12.5 h. Thus, the timing of the low tide(s) experienced by capuchins shifts approximately 30 min each day (see electronic supplementary material, figure S1 for the timing of low tides for one month). As such, visits to the intertidal zone at a repeatable time each day are insufficient for capuchins to regularly exploit coastal resources.

Here, we investigate how the pattern of Coiban capuchins’ activity varies with shifting tidal cycles, by using camera trap data collected from March 2017 to January 2023. We compare how the patterns of activity, relative to distance from the coast and tidal cycles, differ between tool-users and non-tool-users. As exploitation of intertidal resources likely has a seasonal component (e.g. red deer [49] and baboons [12]), we examine differences in tidal patterns for tool-using and non-tool-using capuchins between the wet and the dry season. Lastly, capuchin activity at the coast can be affected by diurnal activity and space use patterns, which are likely shaped by temperature and sleeping site location. To gain a more complete picture of capuchins' activity at the coast, we also consider intra-diel capuchin activity (i.e. are capuchins more likely to be at the coast at a specific time of day). By jointly examining these questions in a system with tool-using and non-tool-using capuchins, we provide a first exploration of the relationship between tool use and tidal cycles for a non-human animal in a coastal habitat. This provides insights into how an ecological generalist copes with complicated cycles of resource availability in a non-typical habitat, and provides a much needed comparative study to help understand potential hominin behaviour in coastal environments where tool use is unlikely to be preserved [50,51].

## 2. Methods

### 2.1. Subjects and site

Coiba National Park consists of nine islands and over 100 islets located off the Pacific coast of Veraguas Province, Panama. It is a designated UNESCO World Heritage site with endemic animal and plant species [52]. White-faced capuchins (*Cebus capucinus imitator*) live on the islands of Coiba (50 314 ha), Jicarón (2002 ha) and Rancheriá (125 ha). These islands are estimated to have been geographically isolated from mainland Panama for 14 000–18 000 years [53]. The terrestrial mammalian communities in Coiba National Park are depauperate in comparison to forests on the mainland, and mammalian predators are entirely absent. Coiba and Jicarón were used as a penal colony from 1919 until 2004, prior to which the islands were inhabited by indigenous people from 250 CE until about the sixteenth century [54]. In recent years, only the island of Coiba and Rancheriá see constant human occupation at two research stations and a police station; the other islands (including Jicarón) are largely undisturbed by humans in recent centuries [34]. Average annual temperature in Coiba National Park is around 26°C. Rainfall varies seasonally. In the dry season (mid-December to mid-April), there is less than 60 mm of precipitation, while in the wet season there is over 3000 mm of precipitation [55].

Stone tool use on Jicarón has been documented at three types of sites, distinguished by the degree of accumulation of debris and tools due to activity intensity and site erasure [34]: (i) elusive sites with low to no accumulation, such as the intertidal zone where debris is washed away, (ii) sites in streambeds, with low to medium accumulation due to more sporadic tool use, and (iii) high accumulation sites—referred to as ‘anvils’ from here on out—further away from streambeds, where large amounts of debris and tools can accumulate over time as capuchins habitually use tools. Since the start of data collection on Jicarón in 2017, habitual tool use at high accumulation anvil sites has only been observed to occur along an approximately 1 km stretch of coast, likely occupied by a single group of capuchins as documented by camera trap data. In 2022, in-person observations confirmed the occurrence of tool use in the intertidal zone by the two groups neighbouring the approximately 1 km stretch of coast (C. Monteza-Moreno, personal communication 2023). However, extensive camera trapping and surveys of the coast and in riparian areas yielded no evidence of tool use (both at high accumulation anvil sites and streambeds) outside of this stretch of coast. Therefore, we refer to the other surveyed groups on the island as non-tool-using groups.

### 2.2. Data collection and processing

#### 2.2.1. Data collection

We analysed images and videos collected using unbaited camera traps on the island of Jicarón between 25 March 2017 and 24 January 2023 (with a gap in data collection in 2020 due to the COVID pandemic) in 11 deployments of about approximately four months (three to six months). Both still (Reconyx Hyperfire HC600 & HF2X) and video (Reconyx Ultrafire XR6 & XP9) camera traps were used. Camera traps are not fully non-invasive, as they are visually incongruent with the surroundings, and produce sounds or light which may disturb animals (and some individuals/species more than others) [56]. We purchased infrared rather than white flash camera traps to minimize disturbance to the animals.

Still-image camera traps recorded 10 images per trigger event without any between-trigger delays (approx. 1 s between images). Video cameras recorded over a 24-h period and captured one image and a dynamic video per trigger, which means that the camera trap stops recording after 3 s of inactivity, and retriggers if additional movement is detected within 27 s. This results in videos of varying lengths, with a maximum length of 30 s (however, we deployed two video cameras with a static video length of 30 s per trigger). We surveyed 61 camera sites within the range of the Jicarón tool-using group, of which 11 targeted anvil sites, 26 were placed at untargeted locations in the streambeds and forest interior and 24 were placed as a 100-m spaced grid. We also surveyed 43 other camera sites in non-tool use areas on Jicarón, of which 18 were placed at untargeted locations, and 25 in a 100-m spaced grid comparable to the one in the tool-using group's range.

We can reliably identify most members of the tool-using group (due to their unique tool-using behaviour and increased sampling and coding efforts), allowing us to be confident about which cameras were placed in the tool-using group range. For the non-tool-using capuchins, based on co-occurrence of identifiable individuals we are confident that the 25 grid cameras all capture one non-tool-using group. However, the 18 untargeted cameras capture multiple non-tool-using groups due to their spread across the island (see electronic supplementary material, tables S1 and S2 for details on camera trap deployments). Table 1 details our estimates of size and composition of the tool-using group and non-tool-using group sampled by the grid cameras.

**Table 1.** Estimated average group size and composition of the tool-using group and non-tool-using group sampled by grid cameras throughout our sampling period. Estimates are based on a) identifiable individuals, b) the maximum number of capuchins observed in a sequence, and c) the maximum number of individuals of a particular age-sex class observed together. No exact numbers are known because of the nature of data collection via camera trapping and fluctuation throughout our sampling period.

estimated numbers	tool-using group	non-tool-using group
adult females	5–6	5–6
adult males	5–6	5–6
subadults	2–4	2–3
juveniles	6–10	7–9
total	20–25	19–23

In total, 137 cameras (117 stills and 20 videos) were deployed during the accumulated sampling period at 104 sites. Out of these deployments, 89 had a single sampling period. The remaining 15 sites were repeatedly sampled (ranging from 2 to 6 deployments in the same location). Average duration of sampling nights per camera was 145.30 (range 9–260), totalling 12 748 sampling nights for the tool-using group and 7152 nights for non-tool-using groups. Camera traps were deployed at various distances from the coast (meaning the coastal vegetation boundary, for more information see below), and grid cameras were placed further inland than other cameras (for the tool-using group non-grid cameras 0.8 m–140.4 m, grid cameras 11.7 m–328.3 m; for non-tool-using groups non-grid cameras 0.5 m–39.2 m and grid cameras 6.1 m–469.7 m). Few camera traps were placed on the beach directly targeting the intertidal zone due to challenges in mounting (i.e. tree availability and seawater) and vandalism.

#### 2.2.2. Coding of images and data processing

Still-images were compiled into sequences based on the time between triggers: all bursts of images triggered less than 30 s apart were considered part of the same ‘sequence’. Each video of 30 s was considered a single sequence. All sequences were coded in Agouti, an online platform for archiving and annotating camera trap data [57]. In each sequence, we identified the animal species visible and the number of individuals per species. For analyses, we only considered sequences with capuchins in them (*n* = 22 185). We did not include any 0's in our models because an absence of capuchin detections does not necessarily mean an absence of capuchins (e.g. camera traps do not always trigger fast enough to capture a travelling animal). Our analyses thus focus on comparing differences in patterns of detection rates in capuchin activity rather than the absence or the presence of capuchins. Further, our focus is not to estimate activity patterns as is commonly done for multiple species, using timestamps from camera trap pictures [58–60]. Instead we are modeling the non-random, spatio-temporal patterns of animal detection, which we refer to as *capuchin activity*. We excluded deployment set-up and collection days from all analyses, as on these days the human presence may have altered capuchin behaviour.

Tidal data, i.e. timing of low and high tides, were obtained from http://www.tides4fishing.com for Cébaco island, which lies approximately 90 km from Jicarón, and is used locally by fishermen in the area and digitized into a .csv using the software Tabula (https://tabula.technology). As the intertidal zone is maximally exposed around the peak of low tide, we calculated the time difference between the initial timestamp of each photo sequence and the time of the nearest low tide. These values ranged from approximately −6 to 6 h, with 0 indicating the peak of low tide and around −6 and 6 the peaks of high tides. Negative values indicate times where the tide is receding, positive values indicate that the tides are approaching the coast.

We calculated the distance of each camera site to the coast by taking the distance from a camera's GPS point to the nearest coastal vegetation boundary. To determine the coastal vegetation boundaries, we used high-resolution satellite imagery from Planet Labs [61]. We used 3 m resolution, four-band surface reflectance Planet imagery from 29 January 2021. On this date, cloud cover was near zero and the image was collected at low tide. We used a normalized difference vegetation index threshold of 0.7 to determine the boundary between coastal vegetation and sand or rock. Data were processed in Google Earth Engine and using the terra package in R [62]. We split the data into dry and wet season based on known rainfall and temperature differences, with the months December–April being part of the dry season and May–November being the wet season.

### 2.3. Statistical analyses

We used hierarchical generalized additive models (GAMs) fit using Bayesian regression modelling with Stan via the ‘brm’ function in the brms package v. 2.17.0 [63]. All statistical analyses were done in R v. 4.2.2 [64]. GAMs are extensions of generalized linear models that allow estimation of nonlinear patterns in data without any prior knowledge of the shape of the expected relationship. Inference is done on the basis of a sum of smooth functions, which are penalized regression splines [65,66]. GAMs are especially useful for seasonal and cyclical data where relationships are unlikely to be linear, such as capuchin activity and tidal cycles. While GAMs are well suited for identifying nonlinear patterns in data, they require caution when used for forecasting based on new data [65].

We fit five different GAMs: three including capuchin activity in relation to time to nearest low tide and two considering diurnal activity. For the tidal models, we ran an initial model (model MT_1) on all of the data comparing the effect of tidal cycles on activity between tool-using and non-tool-using groups. Then we considered differences in tidal pattern between the dry and wet season for the tool-using (MT_2) and non-tool-using groups (MT_3) separately. The diurnal activity GAMs examined how spatial spread of activity patterns varies depending on the time of day, also separately for the tool-using (MD_1) and non-tool-using groups (MD_2).

#### 2.3.1. GAM specifications

To compare tool-users to non-tool-users (model MT_1), we fit a Poisson GAM. Our outcome variable was the count of unique capuchins annotated in a sequence. For predictors, we estimated the effects of (i) temporal difference from nearest low tide, (ii) distance of camera trap from coast, and (iii) an interaction of temporal difference from nearest low tide and camera distance from coast. We used tensor product smooths for this interaction, which allows one to model responses of the outcome variable to interactions of multiple variables with different units.

We used a cyclic cubic spline for time to nearest low tide, as 6 h before a given low tide matches up to 6 h after the previous low tide. We also estimated varying smooths of this tensor product for the tool-using and non-tool-using groups, and included if a group were tool-users (1) or non-tool-users (0) as an index variable. As we have uneven sampling between the tool-using and non-tool-using groups, we include a global tensor smoother for distance to coast and time to low tide, and a tensor smoother considering the tool-using group and non-tool-using groups separately as smooth deviations from that overall surface. We included camera trap location as a random effect.

Secondly, to assess seasonality in a possible tidal effect, we fit two more GAMs with the same structure: one for the tool-using group (MT_2) and one for the non-tool-using groups (MT_3). In these models, we estimated varying smooths of the tensor product for the wet and the dry season, and included the season, wet/dry, as a fixed effect.

Lastly, to investigate the relationship between coastal activity and temperature, we used time of day as a proxy, since temperature varies depending on the time of the day. We used a proxy rather than the actual temperature as camera trap measurements of ambient temperature are unreliable and we lack independent measurements (e.g. from a weather station) to validate them. As such, we fit the same structure GAMs as for the seasonality question, one for the tool-using group (MD_1) and one for the non-tool-using group (MD_2). However, we used hour of the day (from 0 to 23) rather than time to low tide.

We *z*-transformed time to low tide, distance from coast, and hour of the day to improve computational speed, model fit, and ease parameter interpretation. All models were fit with mild regularizing priors, using Normal(0,2) for the intercept and estimates, and exponential(1) for standard deviations. We performed a prior predictive simulation to visualize the priors. We ran the final models with three chains, each having 4000 iterations, including a warm-up period of 2000 iterations per chain. Our model was stable with large effective sample sizes (Bulk_ESS and Tail_ESS over 1000 for nearly all estimates) and Rhat values smaller than 1.01. For all models, Pareto *k* estimates were below 0.5. We used the posterior predictive check function to visually assess model fit and confirm our choice of priors. For full model specifications and details, see the electronic supplementary materials and associated reproducible R code.

#### 2.3.2. Assessing reliability of model estimates

Tensor smooth products were visualized into two-dimensional heatmaps (filled contour plots) using the ggplot2 package v. 3.3.6 [67]. To assist in interpretation of these patterns, we used the ‘method of finite differences' to estimate the first derivative of the spline, which allows for identification of periods of change along a fitted spline. Previous implementations of the method of finite differences on GAMs, were done on single-dimension splines in a frequentist context [68,69]. We have built upon this by developing a Bayesian extension of this method for two-dimensional interaction splines [70]. To accomplish this, we first used the ‘posterior_smooths’ function in ‘brms’ to obtain posterior predictions. Then we recomputed posterior predictions after adding or subtracting a small offset, 0.001, for both predictors in the tensor smooth. As predictors were *z*-transformed, 0.001 represents 1/1000th of 1 s.d. for each predictor, a comparable change in both predictors despite their different scales. The first derivative of the two-dimensional tensor smooth can be approximated as follows:

2.1 $$f_{td}(t,d)\;\tilde{\;}\frac{\;f(t+eps_{h},d+eps_{k})-f(t+eps_{h},d-eps_{k})-f(t-eps_{h},d+eps_{k})+f(t-eps_{h},d-eps_{k})}{4*eps_{h}*eps_{k}},$$

where *t* and *d* represent the two predictors in the tensor smooth interaction (e.g. time to low tide and distance to coast). Here *eps_h_* and *eps_k_* represent the small offset that is added or subtracted to each estimate. With the first derivative approximation, we could identify regions of the two-dimensional surface where the slope was non-flat in all four directions represented in the numerator of equation (2.1). If 89% of the posterior uncertainty interval of the first derivative was on one side of 0, then we interpreted it as reliable evidence for a non-zero rate of change. This conservative criterion allows us to identify areas of the two-dimensional surface of model estimates where we have the most evidence that: (i) the model reliably predicts a nonlinear change (ii) this change is consistently (greater than 89% of the time) in the same direction even with small perturbations to the model. Contourplots showing the proportion of the derivatives above and below zero are provided as electronic supplementary material (figures S4, S7, S10, S12 and S14).

All data and code used for analyses in this paper are available via https://doi.org/10.5281/zenodo.8129505 [71].

## 3. Results

Capuchins live at high densities on Jicarón, but use different locations with varying intensity. We observed capuchins at least once on every camera trap within each deployment (sequences with capuchins range: 4–1346). On average, 2 ± 1.51 (range 1–22) capuchins were observed per sequence. All models indicated considerable variation between camera locations (see electronic supplementary material, figures S2, S5 and S8 for model estimates of capuchin activity per camera location).

### 3.1. A guide to interpreting two-dimensional heatmaps of derivatives

**Figure 1.**
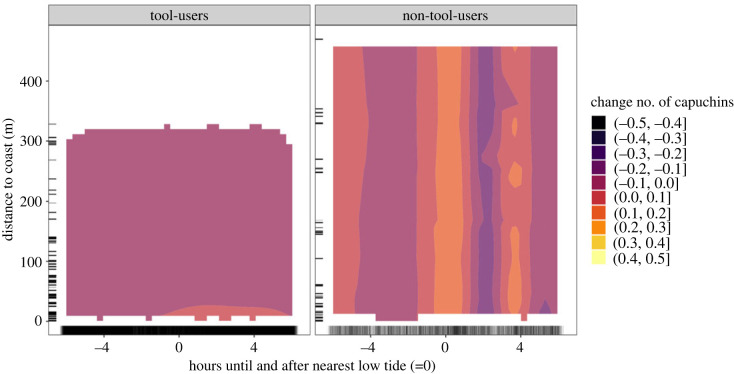
*Tidal activity of tool-using* *versus* *non-tool-using capuchins*. Two-dimensional heatmap showing capuchin activity (colour) at various distances to the coast (*y*-axis) and hours until and after nearest low tide (*x*-axis), for the (*a*) tool-using group and (*b*) non-tool-using groups separately. More colour-saturated areas indicate where greater than 89% of the posterior distribution of the derivative of the tensor smooth interaction lies on one side of 0. Note the rug showing the density of sampling, which reflects the greater density of sampling close to the coast and sparser sampling further inland.

We present the best predictions of our model in figure 1. We guide the reader through how to interpret these types of graphs using figure 1 as an example. In these figures, the *y*-axis represents the distance from the coast in metres. The *x*-axis represents hours until and after nearest low tide, where 0 indicates low tide and the boundaries of the graph around −6 and 6 represent high tides. Observations are indicated in the margins of the axes by translucent grey hash marks. In these heatmaps, colour reflects capuchin activity: 0 is the mean number of capuchins from *z*-score transformed raw data. Light colours indicate a greater number of capuchins than the mean, while dark colours indicate a lower number of capuchins. For example, in figure 1*b*, the orange peak at all distances around low tide (0) indicates highest capuchin activity. The small dark purple trough at 2 h after low tide indicates lowest capuchin activity. We use colour saturation to represent evidence for a reliable nonlinear change in the number of capuchins as represented in equation (2.1). Colour-saturated areas have more than 89% of the derivative being non-zero, indicating that estimated changes in capuchin activity are consistently in the same direction (i.e. positive or negative). In figure 1, no areas are colour-saturated, which shows that we do not see strong, reliable evidence for changes as defined by our 89% criterium. Electronic supplementary material, figure S4 shows exactly how much support there is for specific areas of the two-dimensional heatmap by estimating the mass of the derivative of the posterior on either side of zero. This shows the area with most support is from low tide until 4 h after at less than 50 m from the coast for tool-users (the lighter area in figure 1*a*). However, the proportion of the posterior on one side of 0 here is between 75% and 80%, and thus does not meet our 89% cutoff criterium, which is why it is not saturated.

### 3.2. Tool-users versus non-tool-users (Model MT_1)

The influence of tides on space use patterns differed for tool-using and non-tool-using groups. While we observe little difference between tool-users and non-tool-users in the number of capuchins present per sequence (tool-users: 1.60 [95% CI 1.36–1.95], non-tool-users: 1.65 [95% CI 1.42–1.90]; see electronic supplementary material, table S3 for estimates), the patterns of capuchin activity relative to the tidal cycle vary.

For the tool-using group (figure 1*a*), our model estimates a higher number of capuchins near the coast compared to further inland, although the difference is small (change of less than 0.2, see electronic supplementary material, figure S3 for a zoomed in plot of the area 0–50 m from the coast). We see more capuchin detections close to the coast (0–30 m) at and after the peak of low tide (0–6 h) compared with before (−4–0 h). We do not see reliable evidence (greater than 89%) for a nonlinear change in the number of capuchins, but the increase of capuchins close to the coast after low tide is observed 75–80% of the time, based on the derivative of the tensor smooth interactions (electronic supplementary material, figure S4).

By contrast, for the non-tool-using groups (figure 1*b*), our model estimates larger changes in capuchin activity related to the tidal cycle, but consistent across all distances from the coast instead of concentrated closer to the coast. Based on the heatmap, non-tool-using capuchins show more activity around the peak of low tide, less activity after (around 2 h), and greater activity 4 h after low tide. There are no areas of the heatmap where there is reliable evidence for a change in capuchin activity (electronic supplementary material, figure S4).

### 3.3. Seasonality: tool-using group (Model MT_2)

We considered seasonality in separate models for the tool-using group and non-tool-using groups. Although for the tool-using group we find comparable numbers of capuchins per sequence between the dry and wet season (dry season: 1.54 [95% CI 1.30–1.79], wet season: 1.63 [95% CI 1.49–1.80]; see electronic supplementary material, table S4), the patterns of capuchin activity in relation to the tidal cycle differ.

**Figure 2.**
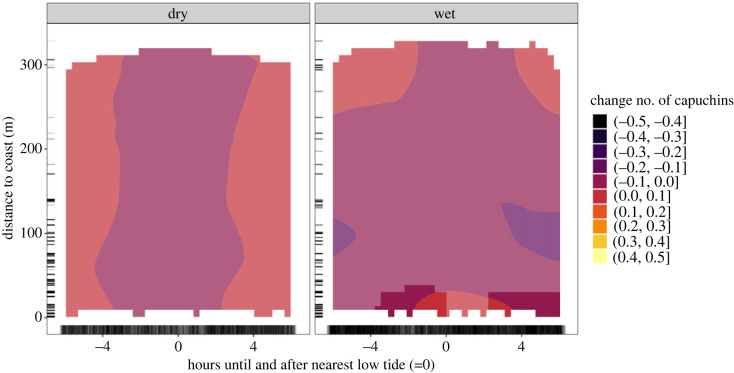
*Tidal activity of tool-using capuchins: dry* *versus* *wet season*. Two-dimensional heatmap showing capuchin activity (colour) of the tool-using group at various distances to the coast (*y*-axis) and hours until and after nearest low tide (*x*-axis), for the (*a*) dry and (*b*) wet season separately. More colour-saturated areas indicate where 89% or more of the posterior distribution of the derivative of the tensor smooth interaction lies on one side of 0.

The activity of the tool-using group varies with the tides throughout the year, but stark differences exist between the patterns observed in the dry and wet seasons. In the dry season (figure 2*a*), capuchin activity at all distances peaks around high tide (−6 and 6 h) and is lowest around low tide (−2 to 2 h). Changes in when and where capuchins are active are smaller in the dry season than in the wet season. Based on the derivative of this model, there are no combinations of distance and time to low tide where we have reliable evidence for differences in activity (electronic supplementary material, figure S7). By contrast, in the wet season (figure 2*b*), we see the opposite pattern. Our model estimates that capuchin activity near the coast is lowest after the peak of high tide, and is highest near the coast immediately preceding and following low tide (−2 to 4 h). Additionally, capuchins are most active further inland (250–300 m from the coast) around high tide (−6 and 6 h). This is also the time period when capuchin activity near the coast is lowest. The derivative highlights large areas where we have reliable evidence for a strong, nonlinear change in capuchin activity, concentrated around the peak of low tide and cameras closer to the coast (less than 30 m). See also electronic supplementary material, figure S6 for a zoomed in view of predicted capuchin activity between 0 and 50 m from the coast.

### 3.4. Seasonality: non-tool-using groups (Model MT_3)

Tidal influence on the space use patterns of the non-tool-using groups is most pronounced during the dry season, which is opposite to our findings for the tool-using group, whose activity near the coast is estimated to peak around high tide in the dry season and around low tide in the wet season. As with previous models, we found no change in the average capuchin activity between seasons (dry: 1.63 [95% CI 1.39–1.92], wet: 1.58 [95% CI 1.39–1.84]; see electronic supplementary material, table S5), but rather changes in the timing of capuchin activity at different distances from the coast.

**Figure 3.**
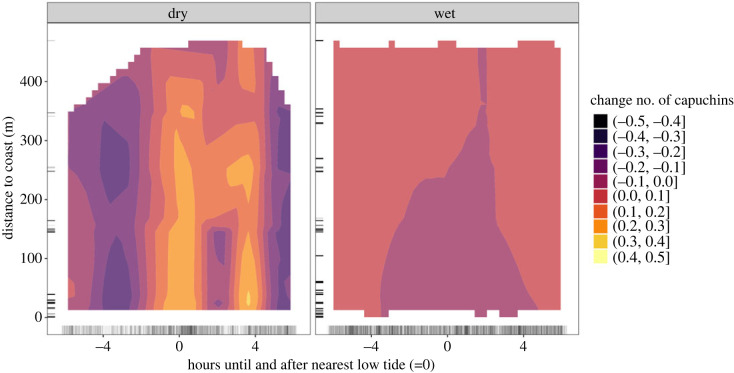
*Tidal activity of non-tool-using capuchins: dry* *versus* *wet season.* Two-dimensional heatmap showing capuchin activity (colour) of the non-tool-using groups at various distances to the coast (*y*-axis) and hours until and after nearest low tide (*x*-axis), for the (*a*) dry and (*b*) wet season separately. More colour-saturated areas indicate where 89% or more of the posterior distribution of the derivative of the tensor smooth interaction lies on one side of 0.

In the dry season (figure 3*a*), the pattern estimated by our model is similar to the predictions of our first model comparing tool-using and non-tool-using groups (figure 1*b*). The model estimates higher capuchin activity near the coast in the hours around low tide (−2 to 2 h, electronic supplementary material, figure S9 for a zoomed in view of the predictions near the coast). The peak of capuchin activity as estimated by the model lies further after low tide and slightly inland (3–4 h after low tide at 20–40 m). We see no reliable evidence for a change in capuchin activity in any regions further of the heatmap (greater than 89%), although at the areas with the largest predicted changes in capuchin activity up to 70–80% of the derivative is on one side of 0 (see electronic supplementary material, figure S10). In the wet season (figure 3*b*), changes in capuchin activity are much smaller than in the dry season. The model estimates a slightly lower number of capuchins near the coast before and after low tide (−4 to 5 h) than around high tide (−6 to −4 and 4 to 6); however, this difference is small (less than 0.1). There are no regions of the heatmap where we have reliable evidence for a change in activity for non-tool-using groups.

### 3.5. Daily activity: tool-using group (Model MD_1)

Consistent daily shifts in temperature or capuchin movement patterns are possible explanations for varying capuchin detection rates at the coast relative to the tides. For the tool-using group, we see a consistently different activity pattern between the dry and wet season in relation to distance to the coast and time of day.

**Figure 4.**
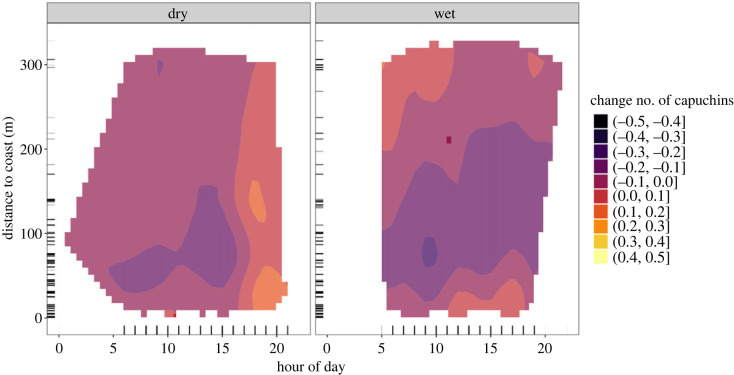
*Diurnal activity of tool-using capuchins: dry* *versus* *wet season.* Two-dimensional heatmap showing capuchin activity (colour) of the tool-using group at various distances to the coast (*y*-axis) and hours of the day (*x*-axis), for the (*a*) dry and (*b*) wet season separately. More colour-saturated areas indicate where 89% or more of the posterior distribution of the derivative of the tensor smooth interaction lies on one side of 0.

In the dry season (figure 4*a*), our model estimates that capuchin activity near the coast is lowest in the morning, and higher only after 17.00 (see electronic supplementary material, table S6 for model estimates). Based on the derivative, we have reliable evidence for a change in capuchin activity in a small region close to the coast around 11.00 (see electronic supplementary material, figures S11 and S12). In the wet season (figure 4*b*), we see higher coastal activity throughout the day, with the peak of activity occurring between 11.00 and 19.00. Before 11.00, capuchin activity inland (greater than 200 m) is higher than coastal capuchin activity. In the dry season, we observe capuchin activity until later in the day (21.00) than in the wet season (19.00). In one small region of the heatmap (approx. 11.00 at approx. 200 m inland), we have reliable evidence supporting a change in the number of capuchins. However, the increase in capuchins' coastal activity in the afternoon is supported with over 80% of the derivative being positive (see electronic supplementary material, figure S12).

### 3.6. Daily activity: non-tool-using groups (Model MD_2)

**Figure 5.**
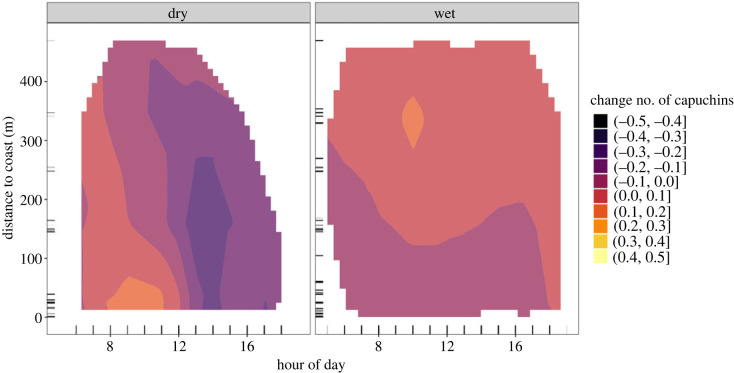
*Diurnal activity of non-tool-using capuchins: dry* *versus* *wet season.* Two-dimensional heatmap showing capuchin activity (colour) of the non-tool-using groups at various distances to the coast (*y*-axis) and hours of the day (*x*-axis), for the (*a*) dry and (*b*) wet season separately. More colour-saturated areas indicate where 89% or more of the posterior distribution of the derivative of the tensor smooth interaction lies on one side of 0.

For the non-tool-using groups, we observed an opposite pattern to the tool-using group when considering activity at varying distances to coast in relation to time of day (figure 5; see electronic supplementary material, table S7). In the dry season (figure 5*a*), our model estimates highest capuchin activity at cameras near the coast (0–70 m) in the morning until 12.00 (see electronic supplementary material, figure S13 for a zoomed in view of predicted coastal activity). Estimated capuchin activity is lowest in the afternoon (13.00–18.00) from near the coast until as far as 300 m inland. We do not have reliable evidence for a change for any areas of the heatmap, but there is up to 85% support for the decrease in capuchin activity in the afternoon (electronic supplementary material, figure S14). In the wet season (figure 5*b*), estimated changes in capuchin numbers are smaller than in the dry season, and there are again no areas of the two-dimensional heatmap where the derivative provides reliable evidence for a change. The model estimates a slightly lower number of capuchins near the coast than further inland throughout the day, until the late afternoon, when capuchins’ coastal activity increases slightly. Highest capuchin activity lies inland (300–400 m) in the morning.

## 4. Discussion

White-faced capuchins living on Jicarón island adjust their activity patterns in correlation with tidal cycles. Our findings are consistent with the hypothesis that availability of intertidal resources plays an important role in shaping patterns of movement and habitat use. We find seasonal variation in the relationship between capuchin activity and the tidal cycle, as well as in capuchins' daily activity patterns. Tool-using and non-tool-using capuchins are seen less frequently near the coast in the dry season during the hottest times of the day (approx. 13.00–17.00), suggesting that high temperatures likely limit capuchins’ activity near the coast and potentially in the intertidal zone. Although capuchins in both tool-using and non-tool-using groups consume intertidal resources, they show different patterns of activity in relation to tidal cycles. Activity of the tool-using group near the coast increases around high tide in the dry season, and around low tide in the wet season. Patterns are more pronounced in the wet than the dry season, when differences in estimated capuchin activity are larger and we have more reliable evidence supporting these changes. By contrast, non-tool-using groups' activity near the coast is higher around low tide in the dry season and around high tide in the wet season, with more pronounced differences in capuchin activity in the dry season.

### 4.1. Capuchin activity varies in relation to tidal cycles

By showing that tool-using and non-tool-using capuchins’ activity at the coast is related to the tidal cycle, we provide additional evidence that exploitation of intertidal resources by maritime foragers shapes the rest of their activity patterns, similar to seabirds [28] and arctic foxes [29]. While from our data, we cannot conclude whether exploitation of the intertidal zone is opportunistic or obligate [5], the tight coupling between Coiban capuchin's coastal activity and tidal cycles indicates that Coiban capuchins likely exploit intertidal resources regularly. Tool-using and non-tool-using capuchins consistently spend time near the coast at either low or high tide, depending on the season. While there are multiple factors that could attract capuchins to the coast (e.g. coastal resources which are not linked to the tides, like coconuts, crabs and sea almonds), because the timing of low tide shifts each day, the observed patterns cannot arise from intermittent activity near the coast irrespective of the tidal cycle. These findings indicate that Coiban capuchins are timing their activity at the coast to coincide with specific parts of the tidal cycle.

### 4.2. Temperature limits coastal activity

Tool-using and non-tool-using capuchins show increased coastal activity at different periods of the tidal cycle (figure 1). To understand these differences, we first have to consider differences in coastal activity between seasons. If seasons are jointly analysed, signatures of seasonal variation in coastal activity can be weakened or masked if a pattern is only present in one season and absent in the other, or if seasonal patterns are opposite to one another. Higher temperatures and lack of rainfall in the dry season affect capuchins' daily activity and, potentially, their exploitation of the intertidal zone. During the hottest times of the day, capuchins prefer resting rather than energy-expending activities like foraging and travelling [72], and presumably, tool use. This is supported by both the tool-using group and non-tool-using groups showing lower activity in the dry season during the hottest time of the day (approx. 15.00) at all distances to the coast (figures 4*a* and 5*a*). Capuchins might compensate for missed daylight activity by staying active later in the evening. We see this for the tool-using group who shows activity up to 2 h later in the dry season than in the wet season (figure 4*b*).

Temperature regulation might also be a driving force to spend time near the coast in general, or at varying moments of the tidal cycle. Overall, the microclimate at the coast is cooler than inland, due to the atmospheric phenomenon of sea breezes which carries cool air to the coast [73]. At high tide, this cooler microclimate would be more pronounced than at low tide, when the water has further receded. While hot temperatures might push capuchins to the coast where it is cooler, heat might also repel them from the intertidal zone. When it is hot, capuchins might be less inclined to venture into the intertidal zone, where there is no shade, the rocks and sands heat up in the sun, and water in tidal pools and crevices evaporates more quickly. This rapid evaporation increases water temperature and salinity, which reduces the number of species that survive in tidal pools. Thus, at high temperatures, capuchins might be attracted to the cooler coast, but avoid the exposed intertidal zone where it is hotter with less available prey.

### 4.3. Seasonality in capuchin activity: tool-users versus non-tool-users

The tool-using group shows decreased activity at all distances from the coast in the dry season around low tide (figure 2*a*), which supports the idea of temperature limiting their (coastal) activity. Immediately after high tide, the sand is still wet and cool from the receding water and the capuchins can remain close to the shade of the tree line, making it possible to consume snails, such as *Nerita scabricosta,* which migrate to the top of the intertidal zone at high tide [74]. However, the lack of differences in activity between coastal and inland cameras suggests this pattern might be more related to temperature regulation aspects of the coast rather than potential intertidal foraging. In the wet season, when temperature is not a limitation, we see reliably high coastal activity of the tool-using group up to 2 h before and 4 h after the peak of low tide (figure 2*b*). This group may exploit the full intertidal range that is exposed in the hours before and after low tide. In contrast, the non-tool-using groups show highest coastal activity in the dry season around low tide and 4 h after low tide (figure 3*a*), which is unexpected if temperature is a major limitation. However, increased capuchin activity is not localized at the coast but rather extends far inland. One possible explanation is that non-tool-using capuchins are at the coast during low tide only for some low tides of the day, while for others they are resting or further inland. Some low tides will be cooler than others: the intertidal zone is likely colder during a low tide at 9.00 than one at 14.00. Non-tool-using capuchins might be near the coast in the dry season when it is not the hottest time of the day, and only venture into the intertidal during these cooler low tides, which could lead to the observed pattern. The non-tool-using groups do not appear to consistently be closer to the coast around low tide, but rather only after high tide (figure 3*a*). Additionally, differences in capuchin activity near the coast are less certain than for the tool-using capuchins, indicating that non-tool-using capuchins show less consistent activity patterns with the tidal cycles.

### 4.4. Seasonality in food availability as a driver of intertidal exploitation

Despite temperature limiting activity in the dry season, this appears to be when non-tool-using capuchins show the strongest tidal pattern (figure 3*a*). The contrasting pattern of tool-using capuchins showing most coastal activity correlated with the tidal cycle in the wet season raises the question: what role does seasonality in food availability play in intertidal exploitation? Resource scarcity is likely experienced differently by tool-using and non-tool-using groups, as tool-using groups can access structurally protected resources more efficiently [17]. Additionally, the tool-using group is located on the side of Jicarón facing the open sea, where exposure effects are more severe and possibly fewer resources are available than at the other side of the island. We have no direct information on seasonal fluctuation in food availability on Jicarón, but tool use occurs more frequently in the transition periods between wet and dry seasons, which might be in response to a limitation in terrestrial resources [34]. This would be in contrast to tool use in *Sapajus* capuchins, which was found to be driven by opportunity rather than necessity [22,75]. It is important to consider that capuchins are dietary generalists who not only consume fruits but also insects and other invertebrates, which have their own seasonal fluctuations. Additionally, oceans are also seasonal and, as such, resources in the intertidal zone likely vary spatially and temporally across seasons (e.g. snails [76]). However, for tool-using capuchins, exploitation of intertidal resources might not be driven by a scarcity of terrestrial resources: in the wet season, there is likely peak availability of one of the tool-users most consumed resources, *Terminalia catappa,* which bears fruit from January to April and from May to September [77]. This coincides with high rates of coastal activity around low tide and the possible exploitation of intertidal resources.

### 4.5. Tool-users versus non-tool-users: why such different patterns?

Why do we observe a difference in the relationship between activity and tidal cycles for the tool-using group and non-tool-using groups? The non-tool-using groups show more activity at the coast during low tide in the dry season, when temperature likely constraints coastal activity, and no clear tidal pattern in the wet season (figure 3). The tool-using group's coastal activity is clearly consistent with exploitation of tidal resources around low tide in the wet season (figure 2*b*). We propose two explanations for why non-tool-using groups might exploit intertidal resources more opportunistically or more rarely than tool-using capuchins.

First, the spatial fixedness of both materials and resources for tool use might drive tool-users to spend more time near the coast. Stones large enough to serve as anvils and hammerstones are more abundant at the coast and the mouth of streams than further inland and may also be prevalent in the intertidal zone itself. Further, important resources, including bivalves, crabs and sea almond trees are restricted to the coast [77]. Because of these constraints, the tool-using group's important foraging spots and anvils are all close to the coast, giving tool-users more opportunities to pick up visual and auditory cues of low tide and more opportunities to exploit tidal resources. Non-tool-using groups, who also forage on sea almonds but only eat the less calorically dense exocarp of ripe fruits and not the nut inside [34] (also see [78] for evidence in another population), likely need to forage more widely for resources and do not have a similar reliance on coastal locations as the tool-using group. Thus, increased proximity to the coast due to reliance on coastal tool use resources might allow and encourage the tool-using group to forage in the intertidal zone more frequently.

A second explanation is that tool use allows the tool-using group to exploit intertidal resources more consistently than the opportunistic foraging by non-tool-using groups. By allowing access to encapsulated resources, percussive tool use can both broaden the diversity of consumable food items (such as tool-using *Sapajus* in mangroves gaining access to snails [30], improving diet quality [79], and increase the efficiency of consuming known resources [17]). Both (or either) of these aspects of tool use could explain why the tool-users might show more intertidal foraging than non-tool-using groups. The costs due to energy investment, time lost foraging elsewhere, and risk of venturing out into the exposed intertidal zone might be outweighed by tool-users' ability to open more resources in the intertidal with greater efficiency than non-tool-users.

Differences in sampling between the tool-using group and non-tool-using groups in spatial and temporal coverage (a small area with dense camera trapping versus a larger area with sparse camera trapping) as well as comparing one group to a multitude of groups may have affected our results. This possible limitation implies that while we can be more confident of the presence of a tidal pattern in the tool-using group, we should interpret the tidal pattern in the non-tool-using groups’ results with more caution. We only have reliable evidence for a change in coastal capuchin activity in relation to the tidal cycle for the tool-using group in the wet season, results of our other models showed more uncertainty. Given this limitation, we can tentatively conclude that for coastal-living capuchins, investing in consistent exploitation of intertidal resources might be a trade-off between nutrition gained and energy and time expended in which using tools could be a decisive factor.

### 4.6. Opportunities for future research

**Figure 6.**
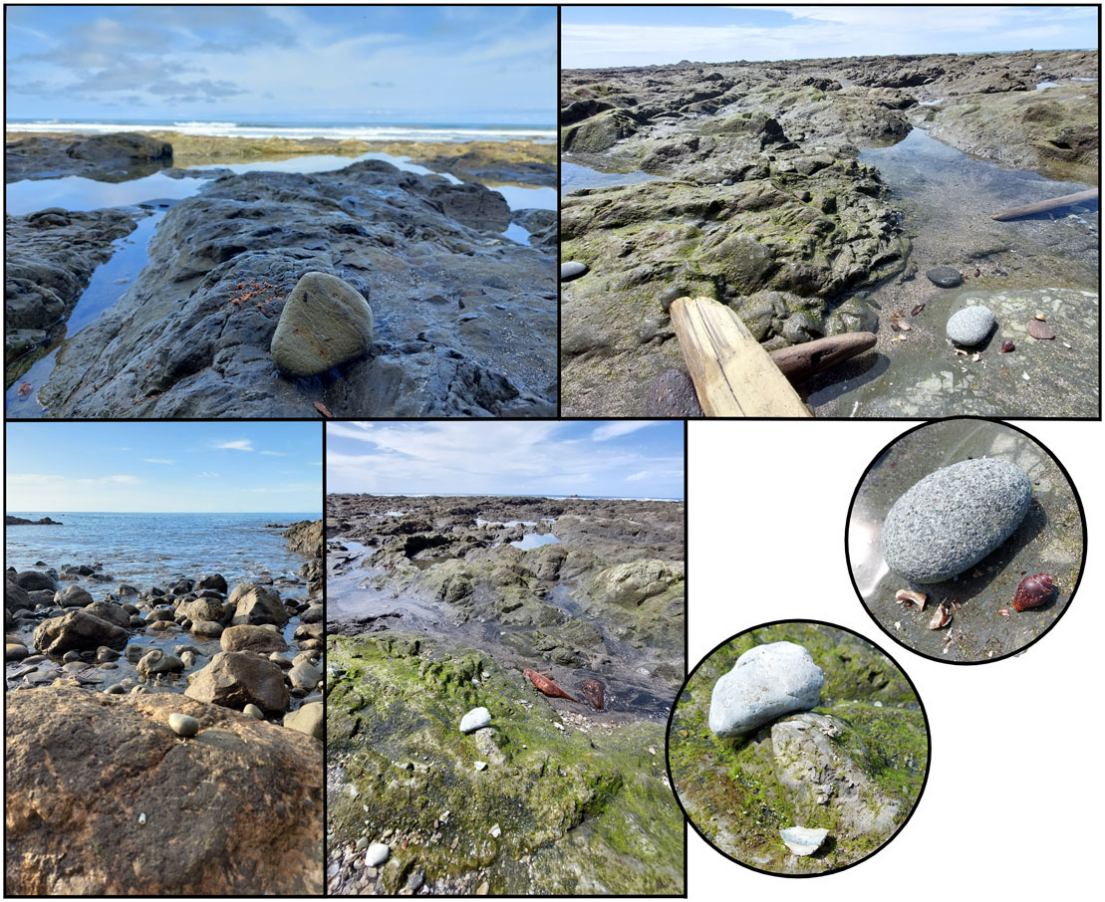
*Evidence of tool use in the intertidal zone*. Hammerstones and debris in the intertidal zone at low tide, which will be washed away by high tide. Photos by Meredith Carlson & Zoë Goldsborough.

To disentangle capuchins visiting the coast for temperature regulation versus foraging on intertidal resources, we need data on capuchin activity from the intertidal zone itself. With camera traps, this was not possible, thus we infer visits to the intertidal based on proximity to the coast. We know that both tool-using and non-tool-using capuchins forage on intertidal resources from in-person observations [34] (B.B., M.C. and Z.G. personal observations), and physical evidence of tool use in the intertidal zone (figure 6). Additionally, capuchins on the island of Coiba also consume intertidal resources like snails [33]. However, more than one process could contribute to the patterns of activity we report, for example, capuchins being near the coast to regulate their temperature. We consider this alternative by including spatial patterns depending on time of day, where time of day serves as a proxy for temperature. Our findings that temperature likely constrains capuchin activity near the coast, in particular in the dry season, suggest that in future research this should be examined in more detail. One possibility is to directly measure the ambient temperature at varying distances from the coast. One unexplored factor that might repel capuchins from the intertidal zone rather than attract them is noise created by the breaking waves, which also fluctuates depending on the tidal cycle. Such loud noises could disrupt vocal communication [80] between group members, and consequently be another trade-off faced by capuchins foraging in the intertidal zone.

Additionally, with semi-arboreal species such as capuchins, terrestrial camera traps miss activity: we cannot distinguish an absence of capuchins from capuchins present but in the trees. While the capuchins on Jicarón are highly terrestrial [45], we attempted to further mitigate this concern by focusing solely on fluctuations in the number of capuchins present in a sequence, rather than drawing conclusions based on the absence. We did not place all of our camera traps randomly, but some were placed in targeted locations, such as on anvils or streambeds, as this study is part of research on tool use. By only comparing fluctuations in capuchin activity *within* a specific camera location we account for this (and other) variation present between different camera locations.

Our findings of such clear tidal patterns in capuchin activity raise the question of how capuchins are aware of the timing of the tides: do they rely on sensory cues (e.g. auditory, visual or olfactory), cues from other species (i.e. seabird activity) or perhaps even cognitively ‘track’ the tidal cycle? Movement data would provide insights on the exact timing of capuchins' visits to the coast, and what underlying mechanism(s) allow(s) them to be there at the right time. Additionally, as the spatial scale of this study (0–400 m from the coast) is limited in comparison to typical capuchin home range sizes (e.g. 0.8–1.5 km^2^ on Barro Colorado Island [81]), tracking of individuals would also allow for integration of our findings into a more comprehensive understanding of Coiban capuchin space use.

There are additional avenues to examine differences in consumption of intertidal resources by the tool-using group and non-tool-using groups, such as DNA barcoding or stable isotope analysis to indicate what proportion of the capuchins’ diet is marine resources (*sensu* [82]). Additionally, a more detailed examination of the variation in daily activity between the tool-using group and non-tool-using groups is needed to explore the effect tool use might have on their behaviour aside from allowing access to tidal resources. Lastly, taking a similar approach in other tool-using and non-tool-using maritime mammals would shed light on whether tool use is indeed important for consistent exploitation of intertidal resources.

### 4.7. Broader implications of our findings

We found that capuchins living in coastal ecosystems show changes in their activity near the coast that correspond to tidal cycles. While both tool-using and non-tool-using capuchins show tidal patterns that correspond with potential exploitation of intertidal resources, only tool-using capuchins show a strong, reliable pattern of increased coastal activity around low tide which is limited to the wet season. Our findings suggest that tidal resources may be very important for coastal-living capuchins, but that tool use might be key, or even a prerequisite, for their efficient exploitation. Although we lack mainland data for comparison, it is important to consider that Coiban capuchins in this study live in a coastal, *insular* habitat. Islands are often resource-limited: species richness decreases with island area and distance to mainland or insular source populations. Additionally, habitual reliance on tool use is observed in many endemic island-living species (i.e. Woodpecker finches [*Cactospiza pallida;* 83], New-Caledonian crows [*Corvus moneduloides*; 24], Hawaiian Crows [*C. hawaiiensis*; 84] and Keas [*Nestor notabilis*; 85]), or restricted to populations that live on islands (i.e. Burmese long-tailed macaques on islands in the Andaman Sea [86]). Potentially, the presence of such a rich intertidal zone in an otherwise challenging ecosystem might be one explanation for why tool use is more likely to arise on islands: to aid exploitation of the intertidal zone.

Percussive tool use on coastal resources also played an important role in human evolution. Prehistoric *Homo sapiens sapiens* used stone tools to access a variety of marine resources from the intertidal zone [87–89]. Consumption of marine resources is argued to have aided the rapid increase in brain size observed in human evolution, due to their high contents of docosahexaenoic acid as well as iodine and selenium [90,91]. Modern coastal-living humans do still employ tools to forage in the intertidal zone, and have been found to prefer foods that provide the best nutritious value for the least effort [92]. Ethnoarchaeological work in humans has shown that intertidal foraging is a high-reward type of foraging—*if* the tidal conditions are optimal [93]. Non-human primates like white-faced capuchins provide an interesting parallel to understand the role tool use has played in human evolution, in the absence of clear evidence from the fossil record.

The findings of this study have implications for the conservation of these capuchins, namely highlighting the potential importance for exploitation of tidal resources for both tool-using and non-tool-using capuchins. Exploitation of intertidal resources by tool-using capuchins appears to be less frequent in the dry season, and due to anthropogenic climate change these periods of hot, dry weather are globally becoming longer and more intense [94]. Further, the ocean is becoming increasingly acidified, which negatively affects many marine organisms [95] and as such will eventually affect the capuchins too as they forage on these marine resources. The role intertidal zones play in capuchins' foraging patterns should be included when considering how to best conserve the capuchins on Jicarón and their unique tool use behaviour [96].

Overall, our findings both uncover the impact exploitation of tidal resources may have on an animal's general activity pattern, as well as increase our understanding of how tool use may provide access to a new niche. Animals that are dietary generalists can opportunistically complement their diet with valuable resources from the intertidal zone, and tool use might be necessary to make the investment in these fluctuating resources worth the costs of keeping up with the tidal cycle.

## Data Availability

All data and code used for analyses in this paper are available via https://doi.org/10.5281/zenodo.8129505 [71]. The data are provided in electronic supplementary material [97].
